# Supplementary material for: C4d Deposition after Allogeneic Renal Transplantation in Rats Is Involved in Initial Apoptotic Cell Clearance
Source: Cells. 2021 Dec 10;10(12):3499. doi: 10.3390/cells10123499 (PMC8700759; doi:10.3390/cells10123499)
Supplement: Supplementary file 1 [file cells-10-03499-s001.zip › cells-1484055-supplementary.pdf]

Table S1. Accession no, gene names and gene symbols of genes used for the validation by the real time PCR.

RefSeq	Gene Name	Gene Symbol
NM_001008515.1	complement component 1, q subcomponent, A chain	C1qa
NM_019262.1	complement component 1, q subcomponent, B chain	C1qb
NM_172222.2	complement component 2	C2
NM_016994.2	complement component 3	C3
XM_001079130.2	complement component 5	C5
NM_022257.1	mannan-binding lectin serine peptidase 1	Masp1
NM_172043.1	mannan-binding lectin serine peptidase 2	Masp2

Table S2. Functional data. Effects of treatments on whole animal functional data (postoperative day 4).

	CTR	sTX	aTX
**Weight loss p.o. (% BW)**	**-**	**3 ± 1 ***	**11 ± 1 *+**
**Urine volume (ml/24 h)**	**14 ± 1**	**20 ± 3 ***	**31 ± 4 *+**
**Na^+ in serum (mmol/L)**	**140 ± 1**	**142 ± 2**	**138 ± 1**
**K^+ in serum (mmol/L)**	**5.6 ± 0.4**	**5.3 ± 0.2**	**5.9 ± 0.5 +**
**FE_{Na+} (%)**	**0.44 ± 0.09**	**0.54 ± 0.11**	**1.2 ± 0.25 *+**
**FE_{K+} (%)**	**28 ± 6**	**34 ± 7**	**49 ± 5 *+**
**CrCl (ml/min/100g BW)**	**0.79 ± 0.04**	**0.34 ± 0.11 ***	**0.32 ± 0.05 ***

Mean values ± SEM, n=5; BW: body weight; p.o.: post operation; CrCl: creatinine clearance; FE_{Na+} and FE_{K+} indicate fractional excretion of Na^+ and K^+, respectively. sTX: syngeneically transplanted; aTX: allogeneically transplanted, *significantly different to CTR ($p < 0.05$), $^+$ significantly different to sTX ($p < 0.05$).

Table S3. Differentially expressed genes associated with the complement pathway. Genes differentially expressed after aTX compared to sTX are listed here (p<0.05).

Gene symbol	Gene name	Fold change
**C2**	complement component 2	**18.3**
**C3**	complement component 3	**10.5**
**C6**	complement component 6	**5.8**
**C1qa**	complement component 1, q subcomponent, A chain	**4.4**
**C1qb**	complement component 1, q subcomponent, B chain	**5.5**
**C1qc**	complement component 1, q subcomponent, C chain	**4.9**
**C1r**	complement component 1, r subcomponent	**3.4**
**C1s**	complement component 1, s subcomponent	**3.1**
**C4a**	complement component 4A (Rodgers blood group)	**2.2**
**C4bpa**	complement component 4 binding protein, alpha	**51.3**
**C4bpb**	complement component 4 binding protein, beta	**7.7**
**C5ar1**	complement component 5a receptor 1	**4.2**
**C8g**	complement component 8, gamma polypeptide	**-2.1**
**Cd59**	CD59 molecule, complement regulatory protein	**-1.4**
**Cfb**	complement factor B	**14.5**
**Cfd**	complement factor D (adipsin)	**2.7**
**Cfh**	complement factor H	**-2.0**
**Masp1**	mannan-binding lectin serine peptidase 1	**1.7**
**Serping1**	serpin peptidase inhibitor, clade G (C1 inhibitor), member 1	**2.3**

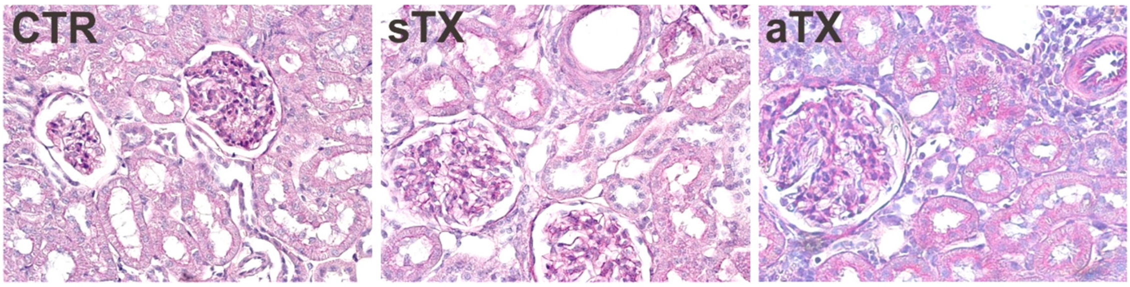

Figure S1. Representative Periodic acid–Schiff (PAS) staining of a control kidney (CTR), syngeneic (sTX) and allogeneic graft (aTX) 4 days after surgery. While histological signs of rejection were absent in CTR and sTX, signs of acute rejection, namely glomerulitis, tubulitis, endothelialitis and graft infiltration were present in aTX. Notably, leukocytes were frequently found adherent to the vessel wall of allografts, whereas vessels of CTR and sTX presented with significant lower numbers of leukocytes.

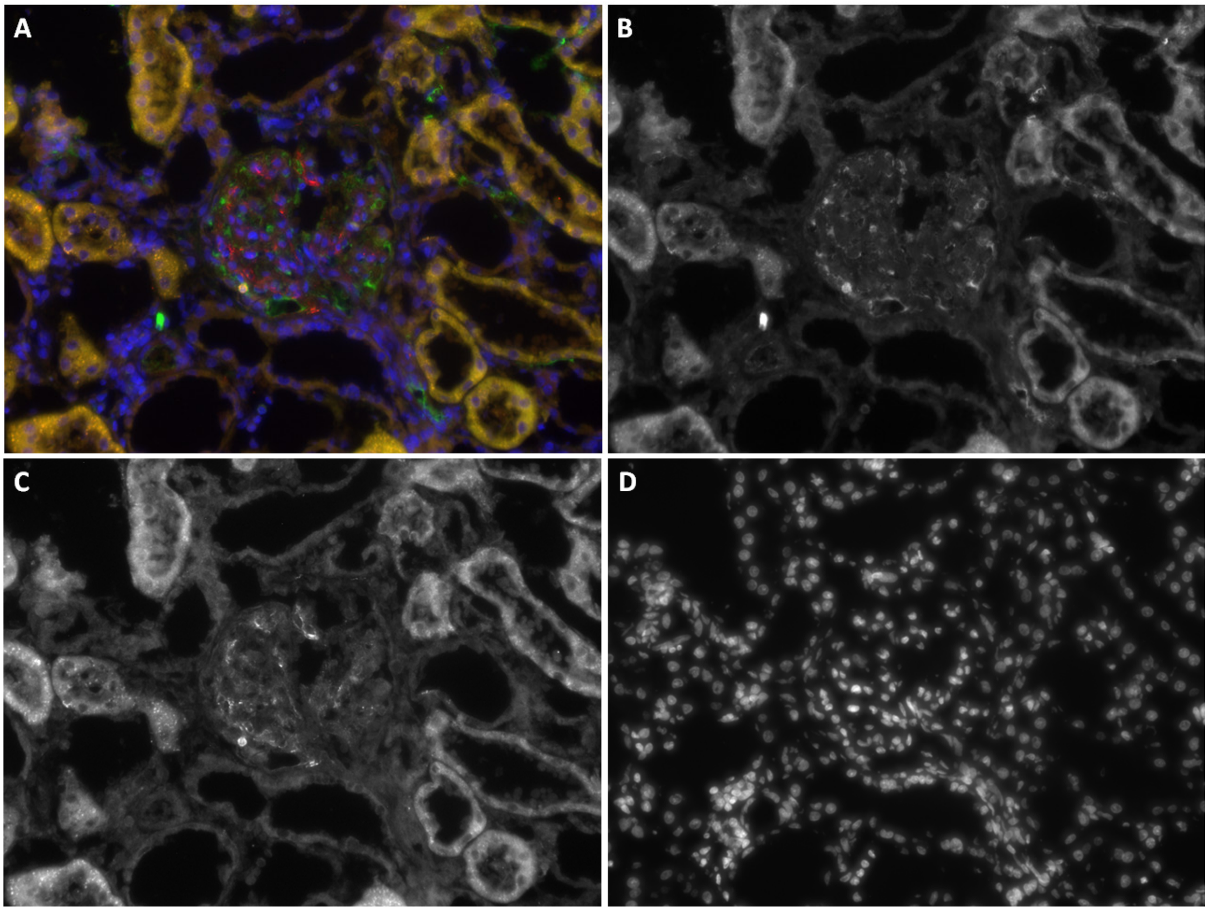

Figure S2. Representative immunohistochemical staining for C4d and IgM on day 1 after aTX. A shows merge image from B-D. B shows staining of C4d in kidney section on day 1 after aTX. After incubation with a C4d antibody and an Alexa-488 labeled secondary antibody signals were visualized by an epifluorescence microscope. C shows staining with an anti-IgM antibody after incubation with Alexa-568 labeled secondary antibody. D shows staining of the nuclei with DAPI.

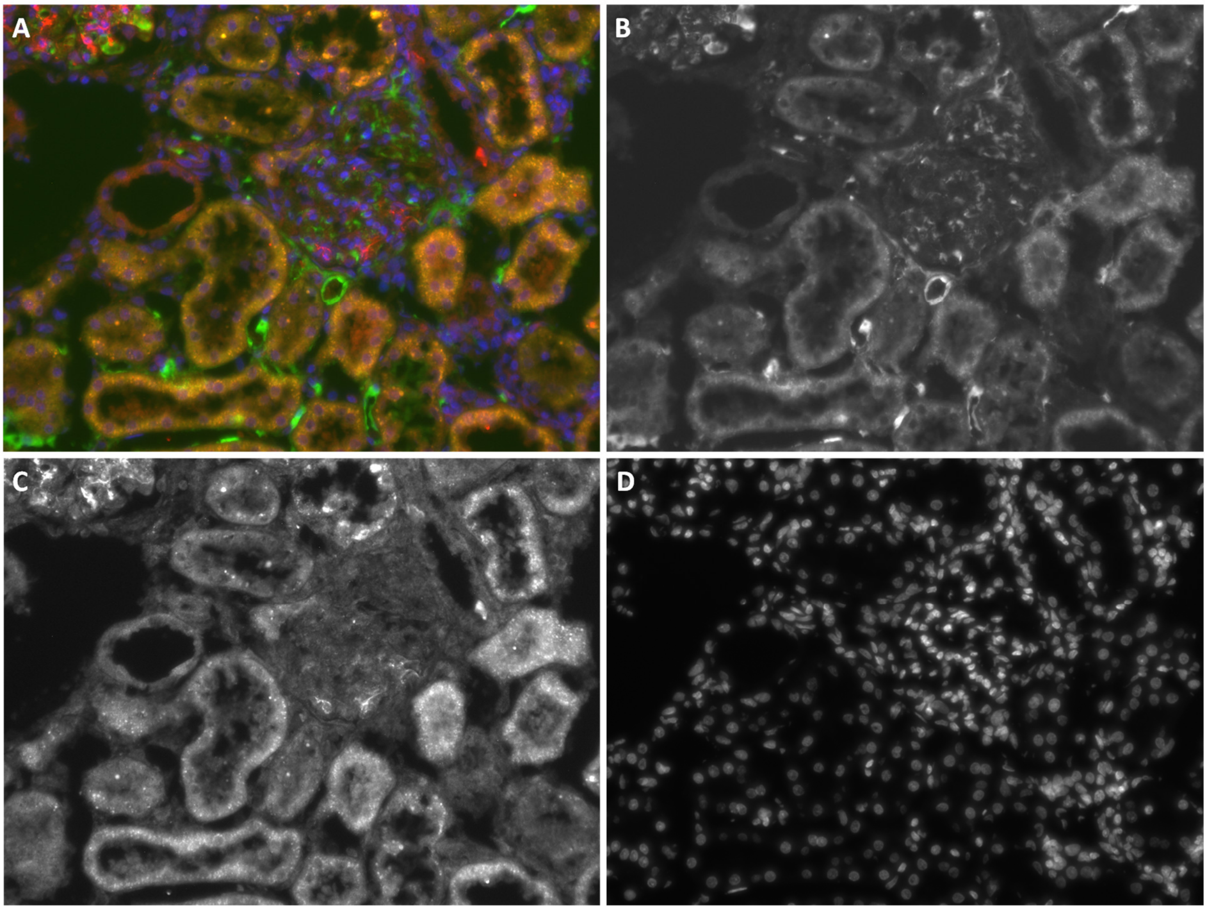

Figure S3. Representative immunohistochemical staining for C4d and IgM on day 2 after aTX. Similar to supplemental figure 2, A shows merge image from B-D. B shows staining of C4d in kidney section on day 2 after aTX. After incubation with a C4d antibody and an Alexa-488 labeled secondary antibody signals were visualized by an epifluorescence microscope. C shows staining with an anti-IgM antibody after incubation with Alexa-568 labeled secondary antibody. D shows staining of the nuclei with DAPI.

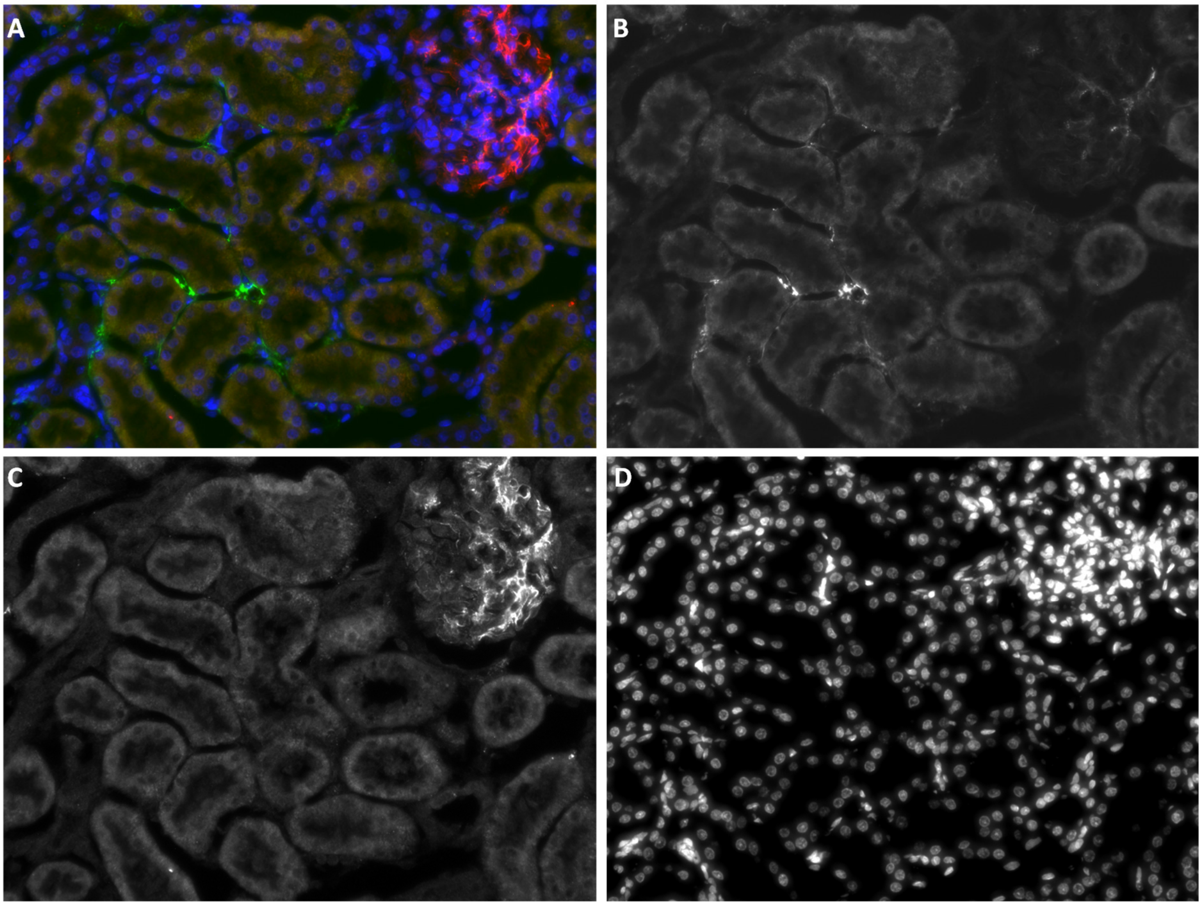

Figure S4. Representative immunohistochemical staining for C4d and IgM on day 4 after aTX. Similar to supplemental figure 2, A shows merge image from B-D. B shows staining of C4d in kidney section on day 4 after aTX. After incubation with a C4d antibody and an Alexa-488 labeled secondary antibody signals were visualized by an epifluorescence microscope. C shows staining with an anti-IgM antibody after incubation with Alexa-568 labeled secondary antibody. D shows staining of the nuclei with DAPI.

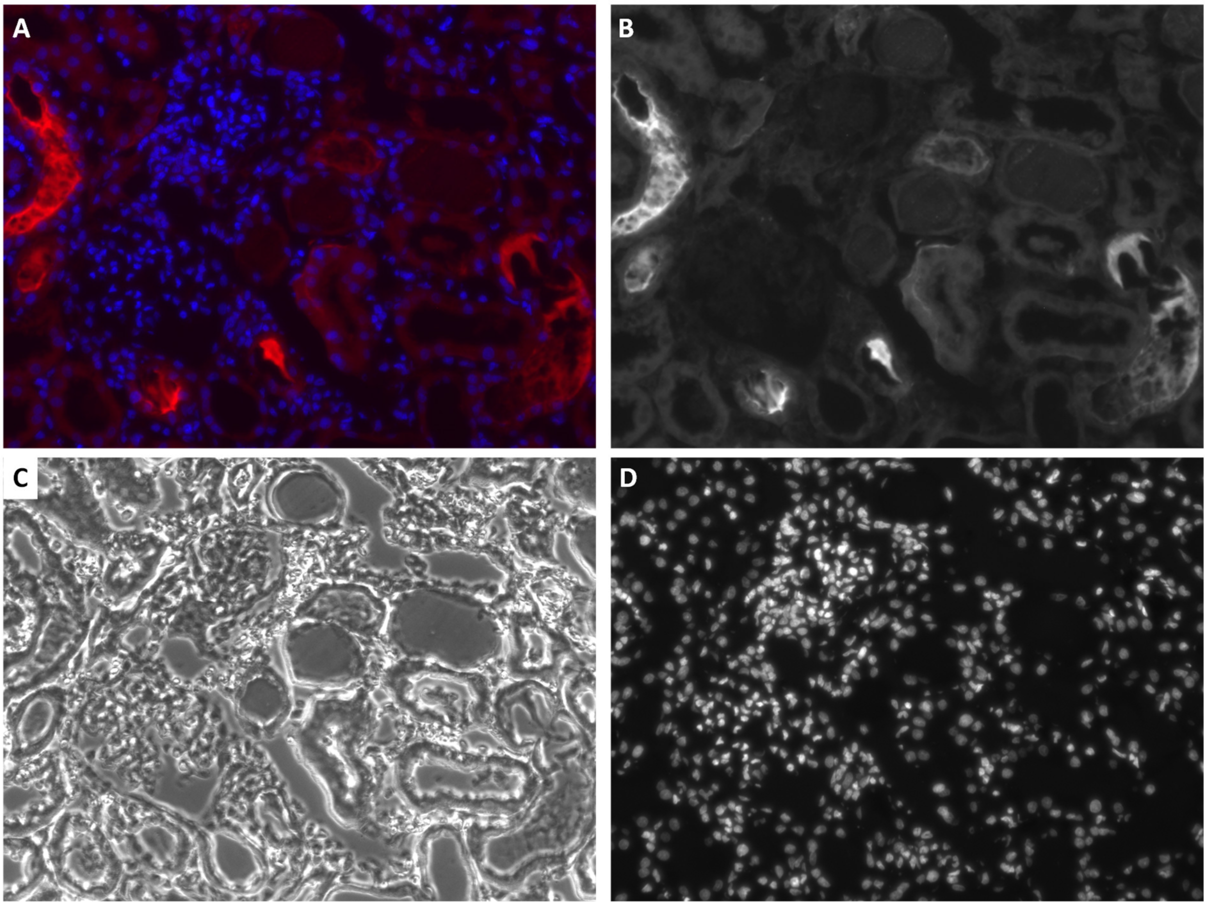

Figure S5. Representative immunohistochemical staining for C4d on day 4 after aTX. Similar to supplemental figure 2, A shows merge image from B and D. B shows staining of C3d in kidney section on day 4 after aTX. After incubation with a C3d antibody and an Alexa-568 labeled secondary antibody signals were visualized by an epifluorescence microscope. C shows brightfield image. D shows staining of the nuclei with DAPI.

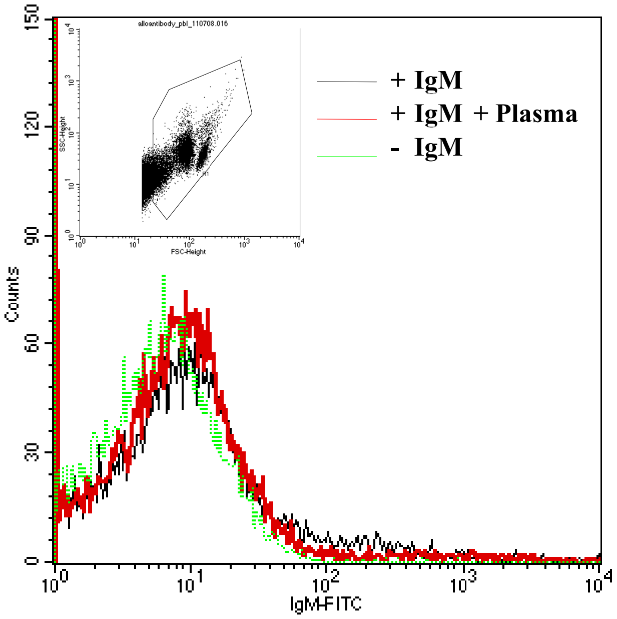

Figure S6. No alloantibodies were detected on day 4 after allogeneic Table 7. Representative immunohistochemical staining for CD55 on day 4 after aTX. Similar to supplemental figure 5, A shows merge image from B and D. B shows staining of CD55 in kidney section on day 4 after aTX. After

incubation with a C55 antibody and an Alexa-568 labeled secondary antibody signals were visualized by an epifluorescence microscope. C shows brightfield image. D shows staining of the nuclei with DAPI.

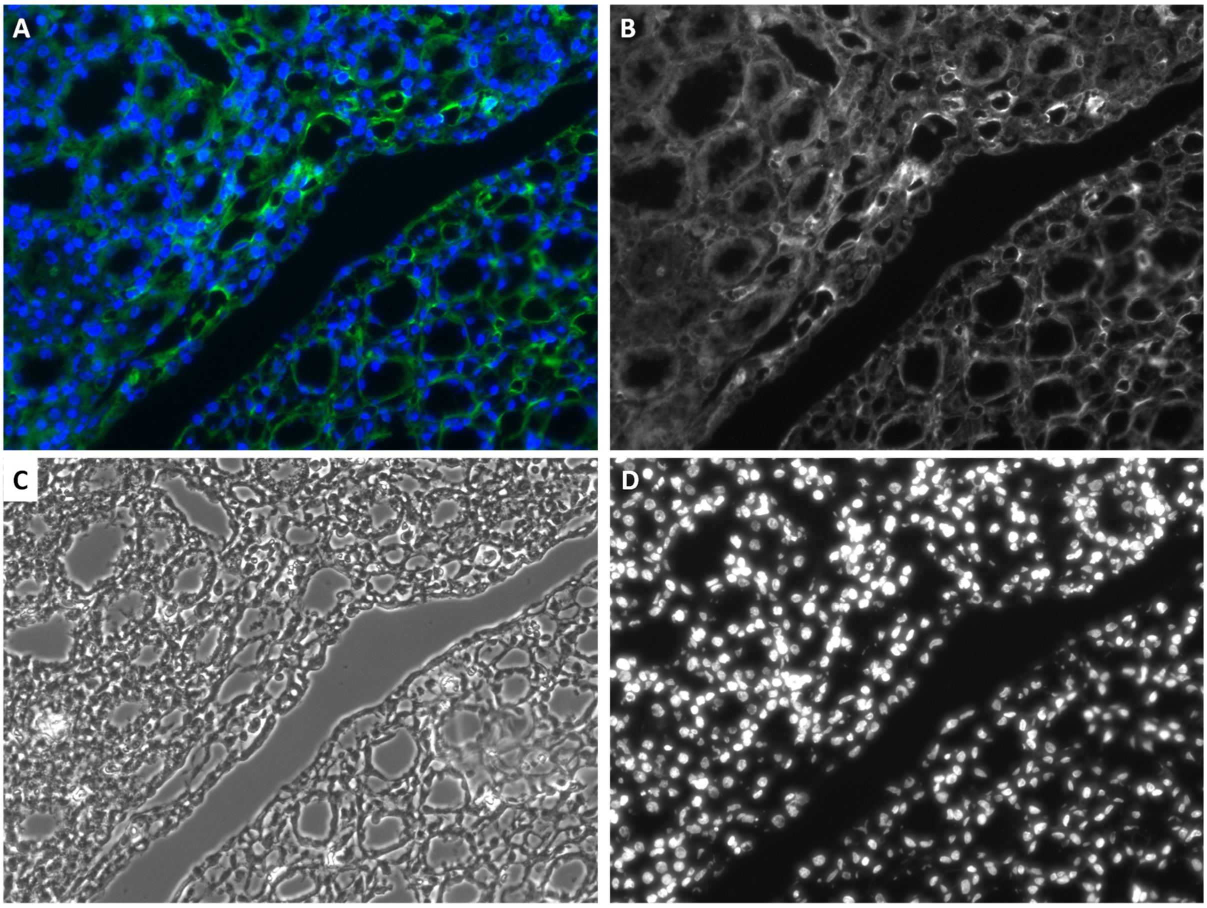

Figure S7. Representative immunohistochemical staining for Crry on day 4 after aTX. Similar to supplemental figure 5, A shows merge image from B and D. B shows staining of Crry in kidney section on day 4 after aTX. After incubation with a Cryy antibody and an Alexa-488 labeled secondary antibody signals were visualized by an epifluorescence microscope. C shows brightfield image. D shows staining of the nuclei with DAPI.
